# Drought-associated genes exhibit high constitutive expression in *Quercus douglasii*, a drought-tolerant California oak

**DOI:** 10.1093/g3journal/jkaf293

**Published:** 2025-12-12

**Authors:** Stephanie E Steele, Lily D Peck, Victoria L Sork

**Affiliations:** Department of Ecology and Evolutionary Biology, University of California Los Angeles, Los Angeles, CA 90095-7239, United States; Department of Ecology and Evolutionary Biology, University of California Los Angeles, Los Angeles, CA 90095-7239, United States; Department of Ecology and Evolutionary Biology, University of California Los Angeles, Los Angeles, CA 90095-7239, United States; Institute of the Environment and Sustainability, University of California Los Angeles, Los Angeles, CA 90095-1496, United States

**Keywords:** differential gene expression, drought tolerance, gene ontology, plasticity, protein families, RNA-seq, transcriptomics, tree, up- and downregulation

## Abstract

Drought stress is a strong selective pressure for all plant species. Plants respond to water shortage through various strategies that confer drought tolerance. These strategies may be plastic responses that occur with the onset of stress or may comprise continuously expressed (constitutive) traits regardless of water availability. Here, we used RNA-seq to characterize transcriptional responses to dehydration in seedlings of a drought-tolerant oak, *Quercus douglasii*, from a local population in the Sierra Nevada Foothills in California. In the greenhouse, we subjected 24 seedlings from 6 maternal families to dry-down or well-watered treatments and prepared RNA libraries from tissue collected before and after each treatment (48 libraries). Our goals were to characterize the pattern of up- and downregulated genes in response to dehydration and to assess the extent to which this drought-tolerant species shows differential versus constitutive expression as a drought response strategy. We identified few differentially expressed genes in response to dehydration. Upregulated genes were related to known drought response functions, while downregulated genes were enriched for gene ontology terms related to growth and carbohydrate metabolism. We discovered high constitutive expression of many putatively drought-responsive genes that had been found to exhibit gene expression plasticity in a different oak species, which is drought-sensitive. This novel finding demonstrates the potential for constitutive expression of genes involved in drought stress to provide an additional mechanism of drought tolerance for some tree species, such as *Q. douglasii*.

## Introduction

Drought is a major source of stress for plants and a strong selection pressure on traits to minimize that stress because intermittent availability of water interferes with core functions. Predicted increases in temperature and decreases in precipitation threaten forests globally (Allen et al. 2010), and large-scale regional die-offs due to drought have already been documented for many tree species (Potter 2016; Choat et al. 2018; Restaino et al. 2019). Drought-related mortality may be the result of decreased carbon assimilation and cellular metabolism and increased susceptibility to other stresses, such as disease and insect attack (Bréda et al. 2006; McDowell et al. 2008, 2022; Anderegg et al. 2016). To respond to water limitation, plants have evolved various strategies to avoid or tolerate stress (Chaves et al. 2003; Verslues et al. 2006; Claeys and Inzé 2013). Plant adaptation to drought stress includes physiological adaptations to maintain water balance throughout dehydration that are controlled by the expression of drought-associated genes. Typical physiological responses include stomatal closure, increased root to shoot growth, reduced leaf expansion, accumulation of solutes, cell wall hardening, and synthesis of protective proteins (Dickson and Tomlinson 1996; Chaves et al. 2003; Verslues et al. 2006). Such responses may increase survival in dry environments, but they may also limit vegetative growth (Verslues et al. 2006; Kaproth and Cavender-Bares 2016). One way to understand complex physiological processes is to use whole-transcriptome sequencing to assay the underlying patterns of gene expression.

Gene expression in response to water limitation may be either a plastic response induced by water stress or continuous regardless of water availability. Plasticity associated with physiological response can be determined by differentially expressed genes, where the functional roles of up- and downregulated genes reflect processes that are being turned on or off, respectively. The extent of differential expression can signal the degree of drought tolerance of a species, with fewer differentially expressed genes in drought-adapted than drought-sensitive species (Madritsch et al. 2019; Mead et al. 2025). Other physiological traits may be shaped by high constitutive gene expression and limited plasticity throughout stress treatments. Such constitutive expression, also described as “frontloading” (Barshis et al. 2013; Rivera et al. 2021), has been observed following stress both within and between populations in rice (Hamann et al. 2024), monkeyflower (Preston et al. 2022), and corals (Barshis et al. 2013). Constitutive expression may facilitate rapid responses to stress and be favorable in environments where stress events are common (Barshis et al. 2013; Rivera et al. 2021). In the case of water stress, it is possible that dry environments with frequent drought events may exert strong selection pressure for high constitutive expression of drought-associated genes and reduced gene expression plasticity. These patterns of gene expression may contribute to drought tolerance strategies and explain differences in drought adaptation among plant species.

Oaks represent an excellent genus with which to examine drought tolerance strategies because they include a range of taxa with different levels of drought adaptation (Kaproth and Cavender-Bares 2016). In general, oak taxa occupy diverse habitats of varying water limitation (Abrams 1990), show high morphological plasticity in drought tolerance (Kaproth and Cavender-Bares 2016), and exhibit variety in water use efficiency between populations within the same species (Roussel et al. 2009). In oaks, several studies have identified gene expression plasticity associated with abiotic stress (Torre et al. 2014; Gugger et al. 2017; Madritsch et al. 2019; Mead et al. 2019a). In 2 prior drought stress studies of *Quercus lobata*, sub-populations from different climate regimes showed different patterns of differential gene expression (Gugger et al. 2017; Mead et al. 2019a). Overall, Gugger et al. (2017) reported many more differentially expressed genes with roughly equivalent numbers of up- and downregulated genes, while Mead et al. (2019a) found slightly more up- than downregulated genes with putative roles related to water deprivation, heat stimuli, hormone signaling, and gene expression. Downregulated genes had photosynthesis- and metabolism-related functions. In general, studies exploring gene expression in response to drought have largely focused on differential expression, which will facilitate plasticity in the affected phenotypes. However, Mead et al. 2025 reported higher levels of constitutive gene expression and reduced plasticity in drought response genes in drought-tolerant relative to drought-sensitive oak species. Gene expression studies that analyze patterns of up- and downregulation versus constitutive expression can provide valuable detail on how trees cope with drought stress.

Here, we explore patterns of gene expression in response to experimental drought treatment in an endemic California oak, *Q. douglasii* Hook & Arn (common name, blue oak), a drought-tolerant oak tree in California (Huesca et al. 2021). Uniquely, this study of a drought-tolerant oak analyzes patterns of differentially expressed genes and constitutive expression of drought-responsive genes identified from *Q. lobata*, a drought-sensitive oak species. We identified these drought-responsive genes across the 2 species by functionally annotating each species' predicted proteins and selecting shared families by protein function (Pfams). We defined patterns of gene expression as constitutive if the initial level of gene expression was greater than zero and remained constant after drought treatment. Specifically, we asked 2 questions. (i) What are patterns of differential expression of up- and downregulated genes in response to drought and what are their functional roles? (ii) Does *Q. douglasii* additionally show constitutive expression of drought-associated genes that could mitigate drought stress? We imposed well-watered and dry-down treatments to 24 seedlings germinated from 6 maternal trees (ie 6 families) from a single natural site in California so that we could both test for treatment effects and genetic differences across families. We measured gene expression by sequencing transcriptomes of all seedlings before and after each treatment (48 libraries). Our study demonstrates that constitutive expression of drought-associated genes is one of many ways for *Q. douglasii* to tolerate drought stress.

## Materials and methods

### Study species and sampling site

*Quercus douglasii* Hook & Arn (blue oak) is a deciduous California endemic oak that is widely distributed across the foothills of the Coastal and Eastern Sierra Ranges, and has more coverage and biomass than *Q. lobata* and *Q. agrifolia,* the 2 other major foothill tree oaks of California, often found in drier sites within oak woodland habitats (Pavlik et al. 1995), and on steeper slopes (Knops and Koenig 1994). Phylogenetically, *Q. douglasii* is in the section *Quercus* and closely related to the shrub white oaks, especially *Q. john-tuckerii*, having emerged as a species about the same time as the arrival of a Mediterranean-type climate in California (Kim et al. 2018; Hipp et al. 2020). Thus, *Q. douglasii* evolved much later than other sympatric California tree oaks in section *Quercus*, including *Q. lobata* and *Q. garryana*, which evolved more than 20 MYA, presumably during cooler temperatures and higher summer precipitation (Hipp et al. 2020). *Q. douglasii* is considered a drought-tolerant oak species (Huesca et al. 2021). Overall, blue oak is an ecologically important and a biologically useful study system with which to pose questions surrounding drought tolerance.

**Fig. 1.**
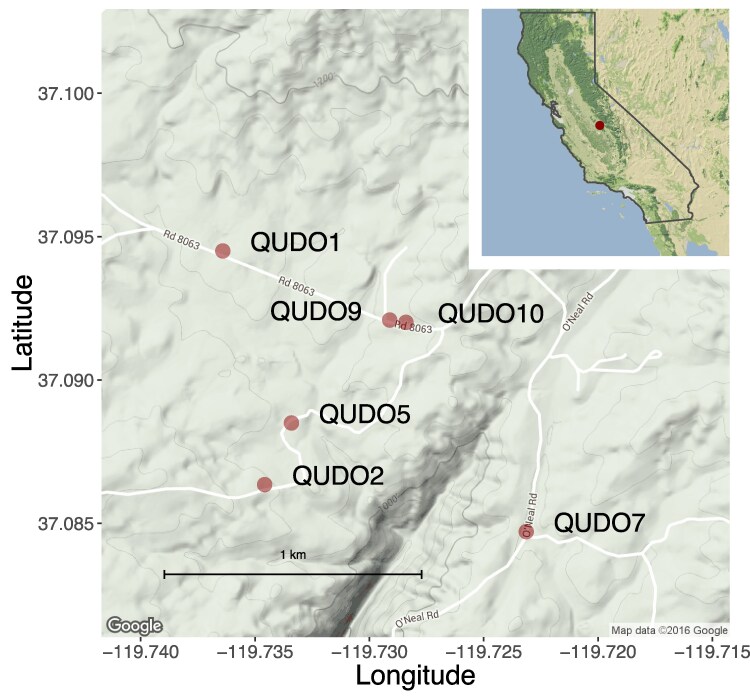
Locations of 6 maternal *Quercus douglasii* source trees whose seedlings provided tissue for the RNA-seq analysis. Maternal trees were located within approximately 1 km^2^ at a single site in the Sierra Nevada Foothills in Madera Co., California. This map was created with the R package ggmap (Kahle and Wickham 2013) using data from Google Maps.

In October 2013, acorns were sampled from 6 *Q. douglasii* adult trees within a single site near the community of O’Neals, Madera County, in the Sierra Foothills in California (37.09201, −119.7284; Fig. 1). Trees were spaced at least ∼50 m apart in this blue oak woodland habitat. We decided to use only a single site to minimize genetic variation and increase our ability to detect a common pattern of gene expression response.

### Experimental design

For each of the 6 maternal source trees, we surface sterilized 10 acorns that were then planted in the greenhouse at UCLA, with an 18-h/6-h light/dark cycle and 20 to 23 °C temperature. Tray positions were randomized every week. After approximately 5 mo in the greenhouse, seedlings were randomly assigned to drought and well-watered treatment groups, with treatment groups and maternal families evenly represented across trays. Well-watered seedlings were watered every few days, while drought seedlings underwent a drought-hardening period for 7 d beginning July 17, 2014, after which they were watered before undergoing another dry-down period for 9 d. The goal of the drought-hardening period was to acclimate plants to water stress, such that a second drought period would capture plant response to dehydration beyond initial shock (Vilagrosa et al. 2003; Villar-Salvador et al. 2004). This approach is intended to simulate natural field conditions in which plants are subjected to repeated drought events. Around the treatment period, seedlings were experiencing infection with powdery mildew. Thus, 2 d prior to the start of the experiment and 7 d into the experiment, seedlings were sprayed with a solution (75 mL dish soap, 300 mL ultrapure 98% petroleum oil in 5 gallons water) to remove powdery mildew. Infection appeared to occur indiscriminately, regardless of treatment group.

### RNA extraction and sequencing

We sampled leaf tissue at 2 time points: time 1 corresponds to day 1 at the start of the experiment at the onset of soil-drying; and time 2 corresponds to day 16 after drought treatment seedlings had undergone 2 dry-down periods. Leaf tissue was frozen between sheets of dry ice in the greenhouse and transferred to a −80 °C freezer. At each of the 2 sampling times, we selected tissue from 24 seedlings from 6 maternal families for RNA extractions, yielding a total of 48 samples. Each maternal family was represented by 2 seedlings per treatment group (soil-drying vs well-watered) at each time point (2 seedlings per family × 6 families × 2 treatment groups × 2 time points = 48 samples). To reduce variation in seedling growth among samples, we selected seedlings from the middle 70th percentile of the height distribution for extractions.

RNA extraction of the 48 samples was conducted as follows. We removed polyphenolics and polysaccharides in leaf tissue using a lithium chloride/urea-based pre-wash protocol developed for conifers (Cronn et al. 2017), which we optimized for *Q. douglasii*. Briefly, 50 to 75 mg of tissue was flash-frozen in liquid nitrogen and transferred to frozen 2 mL grinding tubes with 2 metal beads. Tissue was then ground twice for 1 min at 30 Hz in flash-frozen adapters. We added 1.8 mL cold extraction buffer to each tube consisting of 8 M urea, 3 M LiCl, 1.76% polyvinylpyrrolidone K-60 solution, and 10 mM dithiothreitol (added from 1 M stock just before use). Tubes were vortexed vigorously and ground for 10 s at 30 Hz, followed by centrifugation for 10 min at 1,000 rcf and 4° C that allowed recovery of 1.4 mL supernatant, which was kept at 4° C overnight. The following day we centrifuged tubes for 30 min at 20,000 rcf and 4 °C. After discarding the supernatant, the pellet was washed with 2 rounds of 70% ethanol, each followed by a 5-min centrifugation at 5,000 rcf and room temperature. Finally, the pellet was dried in the fume hood for 10 min. The air-dried pellet was used as the starting material for extraction of total RNA with the RNeasy Plant Mini Kit (QIAGEN, Germantown, MD). An incubation at 56 °C with RLT buffer for 2 min, DNase digestion, and an additional 500 μL wash with buffer RPE increased sample purity and yield. Nine samples with low 260/230 ratios on a NanoDrop ND-1000 spectrophotometer (Thermo Fisher Scientific, Waltham, MA) underwent a bead cleanup using an Agencourt AMPure XP kit (protocol B37419AA; Beckman Coulter, Beverly, MA). Total RNA quality and quantity was verified on a 2100 BioAnalyzer using a eukaryotic total RNA Nano Series II assay (Agilent Technologies, Santa Clara, CA) at the UCLA GenoSeq Core.

We used a TruSeq RNA library preparation kit (Illumina, San Diego, CA) to isolate poly-A tail-selected mRNA and convert to cDNA. Twenty-four unique Illumina adapters were used to barcode individual libraries. Libraries were quantified via a Qubit dsDNA BR assay kit (Thermo Fisher Scientific, Waltham, MA), and average fragment size was estimated on a 2100 Agilent Bioanalyzer using a DNA 1000 Series II assay at the UCLA GenoSeq Core. Samples were then normalized and pooled into 4 libraries, each containing 3 sets of 4 samples representing a drought and well-watered seedling at time 1 and time 2 for a given maternal family. Libraries underwent single-end 50-bp sequencing on 4 lanes of an Illumina HiSeq2000 at the Broad Stem Cell Research Center at UCLA.

### Read processing, transcriptome assembly, and annotation

Raw sequence reads were demultiplexed allowing 1 mismatch per barcode, followed by several filtering steps. Reads that failed the Illumina quality filter were removed, and remaining reads were converted from qseq to fastq format. Scythe version 0.994 BETA (Buffalo 2011) was used to trim adapter sequence, followed by Sickle version 1.33 (Joshi and Fass 2011) to trim low-quality sequence from read ends falling below an average Phred score of 30 in a sliding window and to remove resulting reads less than 20 bp. Low-complexity sequences were removed with the FASTX-Toolkit (Hannon 2010) and processed reads were quality-checked with FastQC (Andrews 2010). To remove ribosomal RNA sequences, reads were mapped to the SILVA rRNA database (Quast et al. 2012) using BBTools “bbmap” with the parameter “outu” to keep only the unmapped reads. This final quality control step removed 8.3 Gb data. The demultiplexed sequence data have been deposited in the International Nucleotide Sequence Database Collaboration (INSDC) database under the BioProject accession number PRJNA1259526.

A de novo transcriptome was assembled using the Trinity platform (Haas et al. 2013). Specifically, we used the genome-guided assembly tool with the *Q. lobata* reference genome, Valley oak genome version 3.0 (Sork et al. 2022) as the closest related species with a high-quality genome and annotation. Lacking a *Q. douglasii* reference genome, *Q. lobata* is sufficiently related to identify drought-response genes to assess trends in gene expression for this study. To functionally annotate the Trinity transcripts and identify candidate coding regions within the transcript sequences of *Q. douglasii*, first we used TransDecoder v5.7.1 (Haas, https://github.com/TransDecoder/TransDecoder). Next, we used Trinotate (Bryant et al. 2017) to build an sqlite database with our sequence data and the TransDecoder output, which we then used to analyze our sequences against the following databases: BLAST (Altschul et al. 1990), Pfam (Punta et al. 2012), GO (Ashburner et al. 2000), and infernal (Nawrocki and Eddy 2013).

### Differential expression and gene ontology analysis

We performed transcript quantification on gene-level abundance estimates in a genome-free way using Salmon v1.5.2 (Patro et al. 2017) on all read libraries. The relationships within and between seedlings for each dataset were visually examined using the “PtR” script in the Trinity toolkit (Grabherr et al. 2011; Haas et al. 2013). The transcript count matrix was tested for differential expression using DESeq2 (Love et al. 2014), which uses negative binomial generalized linear models to test for statistical significance. Differential expression was calculated between time 1 and time 2 separately for drought and well-watered treatments. We also calculated differential expression using edgeR (Chen et al. 2016), which gave similar results (212 differentially expressed genes in DESeq2; 172 in edgeR). We used the Benjamini–Hochberg method (Benjamini and Hochberg 1995) to adjust *P*-values for multiple testing to control the false discovery rate and defined a set of differentially expressed genes with *P*-values <0.01 and log-fold change >2. To obtain a final set of differentially expressed genes, we first removed any genes present in both drought and well-watered treatment groups, and then selected only those significant in both DESeq2 and edgeR.

All differentially expressed genes were classified according to their GO (gene ontology) term, creating enriched and depleted gene classes across the treatment groups. Functional enrichment analysis was run using GO-Seq (Young et al. 2010) with GO terms from Trinotate, through the Trinity toolkit “analyze_diff_expr.pl” with the parameters “–examine_GO_enrichment –GO_annots –gene_lengths.” This script returns differentially expressed genes for enriched and depleted GO categories for the up- and downregulated genes in each comparison. Enriched GO terms were further filtered, using only overrepresented terms with a *P*-value < 0.01 in the analyses.

To test whether maternal families responded differently to drought stress and thus provided within-population variation in drought response, we compared patterns of differential expression by gene and GO term. First, we calculated fold change values (log_2_FC) for each of the 6 maternal families using the equation:


$$\mathrm{lo}g_{2}\mathrm{FC}=\mathrm{lo}g_{2}(\mathrm{mean}\_\mathrm{expression}\_\mathrm{family}\_1\_T2+1)-\mathrm{lo}g_{2}(\mathrm{mean}\_\mathrm{expression}\_\mathrm{family}\_1\_T1+1),$$

where the average expression for a gene or GO term as averaged across individuals within a family, and then averaged within treatment. Second, we tested whether there were differences in expression patterns separately for up- and downregulated genes between maternal families for individual genes with a repeated measures ANOVA in R, using this model: gene expression ∼ treatment | time | family + residual. When family was significant, we then tested for an effect of maternal family using a generalized linear model and specifying a Tukey test, by selecting only drought-treatment individuals at time 2: expression ∼ maternal family + residual. The Benjamini–Hochberg method (Benjamini and Hochberg 1995) was used to adjust *P*-values for multiple testing to control the false discovery rate.

### Comparison of constitutive expression in *Q. douglasii* with *Q. lobata*

To assess whether constitutive expression is a possible drought-tolerant strategy in *Q. douglasii*, we first identified a candidate set of putative drought-responsive genes to examine. The list of genes were those that showed differential expression in *Q. lobata* seedlings in 2 separate studies that were exposed to a soil dry-down treatment, hereafter, “drought-responsive genes,” available through supplementary data files from Gugger et al. (2017) and Mead et al. (2019a). The 2 studies used different cutoffs for genes that exhibited significant differential expression (*P* < 0.05 in Gugger et al. (2017) and adjusted *P*-value < 0.01 in Mead et al. (2019a)), so we simply selected the shared protein families (Pfams) between the 2 studies, resulting in 81 shared Pfam categories to test in *Q. douglasii*. The different cutoffs of the 2 studies do not bias our choice of genes.

To test whether drought-responsive genes were constitutively expressed in *Q. douglasii*, we measured the expression of *Q. douglasii* genes matching the list of Pfams across time 1 and time 2, separately for well-watered and drought treatments. We first controlled for variance in expression levels by scaling the data (dividing by variance in standard deviation), then tested the significance and magnitude of difference using linear models: expression ∼ time | Pfam. We used the R package emmeans (Lenth and Piaskowski 2025) to compute the effect (difference) of time as individual coefficients within each Pfam. We defined gene expression as constitutive when the initial level of gene expression was greater than zero and remained constant after drought treatment.

As a further exploration of the gene expression response of *Q. douglasii* to drought treatment and to distinguish between differential and constitutive gene expression, we conducted a comparison with the drought-sensitive *Q. lobata*. We downloaded the raw sequencing reads from the Mead et al. (2019a) data repository (https://doi.org/10.5068/D1HH31) for 2 *Q. lobata* populations: Centerville, CENT and Malibu Creek, MACR. We selected sites with both similar (Centerville, Sierra Foothills) and contrasting (Malibu Creek, coastal) climates to that of *Q. douglasii* (O’Neals, Sierra Foothills). We performed transcript quantification and differential expression analyses exactly as we did for *Q. douglasii.* To allow comparison across these 3 sites, we analyzed differential expression separately for each *Q. lobata* site and combined the lists of differentially expressed genes.

For this analysis, we selected drought-responsive Pfams present in *Q. lobata* that included at least 2 genes, giving us 20 Pfams, and compared their expression levels to genes comprising the same Pfams in *Q. douglasii.* In our comparison of expression levels of our drought-responsive Pfams in *Q. lobata* and *Q. douglasii,* we first controlled for variance in expression levels by scaling the data. We then tested the significance of overall expression differences between treatments by fitting an ANOVA model: expression ∼ site | treatment, random effect = Pfam. The interaction was significant and so we tested for an effect of site separately for each treatment using a generalized linear model and specifying a Tukey test: expression ∼ site, random effect = Pfam. The Bonferroni method was used to adjust *P*-values for multiple testing to control the false discovery rate. To test the significance and magnitude of expression differences by Pfam, we used linear mixed effect models: expression ∼ treatment, random effect = Pfam.

### Data analysis

All statistical tests were performed in R version 4.4.0 using nlme (Pinheiro and Bates 2025), broom.mixed (Bolker et al. 2024), multcomp (Hothorn et al. 2008), and emmeans (Lenth and Piaskowski 2025). The following R packages were used in analyses and to make figures, all using the latest versions: tidyverse (Wickham et al. 2019), and cowplot (Wilke 2024). This work used computational and storage services associated with the Hoffman2 Cluster, which is operated by the UCLA Office of Advanced Research Computing's Research Technology Group.

## Results

### Differential expression following drought stress in *Q. douglasii*

**Fig. 2.**
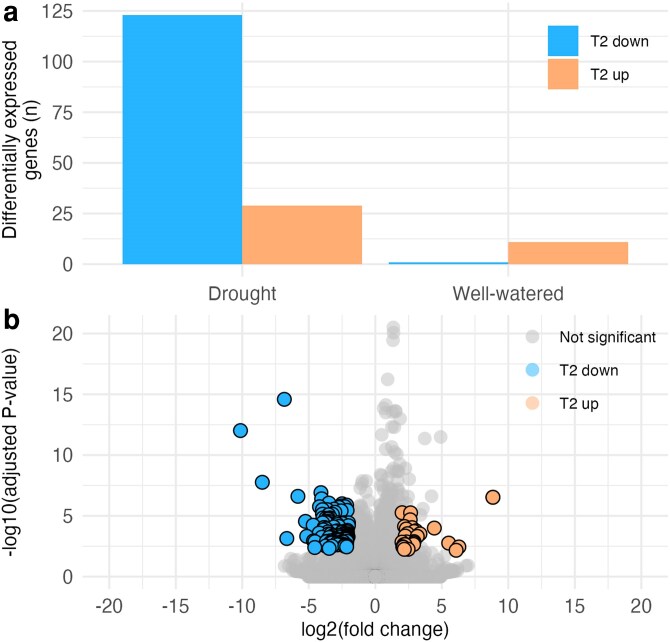
Transcriptional response of *Quercus douglasii* seedlings to experimental drought stress. a) The number of genes showing differential expression, both up- and downregulated, in the drought-stressed and the well-watered treatment group at time 2, as compared with time 1. Values represent genes that were identified as differentially expressed using negative binomial generalized linear models in both DESeq2 and edgeR, and any genes which were differentially expressed in both drought and well-watered treatments were removed. b) Volcano plot showing the total differentially expressed genes at time 2 relative to time 1 in the drought treatment group. Each point represents the log_2_ fold change and −log_10_ (adjusted *P*-value) for a single gene. Orange points (highlighted on right side of plot) are upregulated genes in response to drought; blue points (highlighted on left side of plot) are downregulated genes; gray points are genes that are not significantly differentially expressed.

The 48 RNA-seq libraries yielded 914.3 million total raw reads, reducing to 772.9 million reads following quality control. On average, filtered libraries contained 16.1 million ± 2.57 million reads. Among 50,362 total expressed genes, 152 genes were differentially expressed across the drought time 2 samples in both DESeq2 and edgeR (Fig. 2). Most of these differentially expressed genes were downregulated (123) in the drought-stressed individuals, while a smaller number (29) were upregulated (Fig. 2a). In the well-watered group, just 1 gene was downregulated between Times 1 and 2, while a modest number were upregulated. The log_2_ fold change values of the significant differentially expressed genes were comparable between down- and upregulated genes (Fig. 2b).

### Significant upregulation of drought-associated genes

**Fig. 3.**
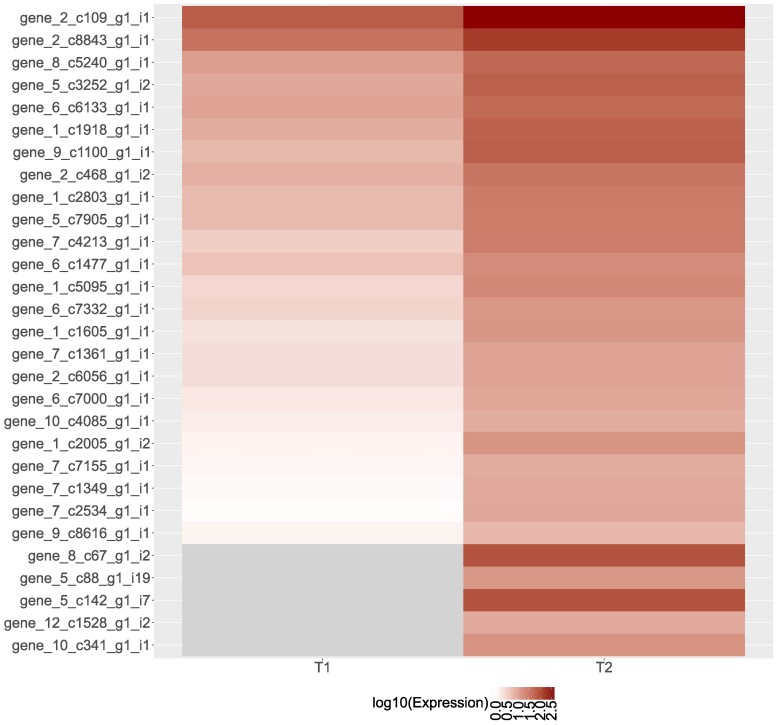
Heatmap of gene expression in 29 significantly upregulated genes following drought treatment. Expression was measured in 12 *Quercus douglasii* seedlings sampled at 2 time points, T1, at the initiation of soil drying treatment, and T2, 16 d following treatment. For each treatment and time point, we averaged expression for each gene by maternal family. None of these genes showed significant upregulation in the well-watered treatment. Log-fold changes varied from no expression (gray) to low (white) to 2.5 (dark red) log_10_ increase in mean expression. See Table 1 for functional annotations.

We found more upregulated genes in the drought-stressed treatment than in the well-watered treatment (Fig. 2) and only 29 genes that were significantly upregulated in the drought-stressed, but not well-watered seedlings (Fig. 2). We did not find an effect of maternal family on expression for upregulated genes (Supplementary Table 1), which means that all seedlings upregulated genes in a similar manner in response to the drought treatment. Of the significant genes, a few changed dramatically from zero or little gene expression to extremely high expression, such as GENE_8_c67_g1_i2 and GENE_5_c142_g1_i7 (Fig. 3), making them good candidates as drought-responsive genes, but we were unable to identify gene function (Table 1).

**Table 1.** Functional annotations for 29 significantly upregulated *Quercus douglasii* genes in response to drought treatment shown in Fig. 3, ranked by significance value.

Gene	BLASTp function	Pfam	log_2_fold-change	Adj-*P*- value
GENE_8_c67_g1_i2			8.842	2.97E−07
GENE_5_c142_g1_i7			8.842	2.97E−07
GENE_2_c8843_g1_i1	Cold-regulated protein 27 [ECO:0000303|PubMed:19566593]		2.016	5.52E−06
GENE_5_c3252_g1_i2	Monooxygenase 1 [ECO:0000303|PubMed:10216258]	PF01494.24, PF01266.29, PF13450.11	2.652	5.65E−06
GENE_2_c109_g1_i1		PF03350.21	2.624	2.10E−05
GENE_6_c6133_g1_i1	Serine acetyltransferase 1, chloroplastic	PF06426.19, PF00132.29 PF14602.11	2.196	7.52E−05
GENE_7_c7155_g1_i1	Transcription factor bHLH96	PF00010.31	2.758	9.30E−05
GENE_1_c2005_g1_i2			4.436	1.01E−04
GENE_10_c4085_g1_i1			2.330	1.21E−04
GENE_7_c4213_g1_i1			3.052	1.69E−04
GENE_1_c1605_g1_i1			2.914	1.93E−04
GENE_1_c2803_g1_i1	NAC domain-containing protein 90	PF02365.20	2.423	2.92E−04
GENE_7_c2534_g1_i1			3.356	3.04E−04
GENE_2_c468_g1_i2	Protein REVERSION-TO-ETHYLENE SENSITIVITY1	PF05608.17	2.249	4.57E−04
GENE_9_c1100_g1_i1			3.155	4.88E−04
GENE_1_c5095_g1_i1			2.908	1.32E−03
GENE_5_c7905_g1_i1			2.347	1.39E−03
GENE_8_c5240_g1_i1	NAC transcription factor 47 [ECO:0000305]	PF02365.2	2.005	1.45E−03
GENE_7_c1349_g1_i1			2.892	1.70E−03
GENE_12_c1528_g1_i2	PXMP2/4 family protein 3	PF04117.17	5.517	1.75E−03
GENE_1_c1918_g1_i1			2.876	2.25E−03
GENE_2_c6056_g1_i1			2.159	2.31E−03
GENE_6_c7332_g1_i1			2.231	2.32E−03
GENE_7_c1361_g1_i1			2.065	3.50E−03
GENE_10_c341_g1_i1	PP2A regulatory subunit TAP46 [ECO:0000303]	PF04177.17	6.284	3.63E−03
GENE_6_c1477_g1_i1			2.110	3.84E−03
GENE_6_c7000_g1_i1			2.468	5.60E−03
GENE_9_c8616_g1_i1			2.179	5.67E−03
GENE_5_c88_g1_i19			6.077	6.81E−03

Among the 29 genes, only 1 GO term comprising multiple genes was enriched: 4 genes were grouped into the enriched molecular function GO term “DNA-binding transcription factor activity.” Because upregulated genes largely did not match GO terms, we instead focused upon BLAST hits. Nine genes returned hits (Table 1), of which many had a putative function associated with drought response. Specifically, these genes included a cold-regulated protein, monooxygenase, serine acetyltransferase, transcription factor bHLH96, NAC domain-containing protein and transcription factor, reversion to ethylene sensitivity protein, and PXMP2/4 family protein.

### Significant downregulation of genes associated with growth functions

**Fig. 4.**
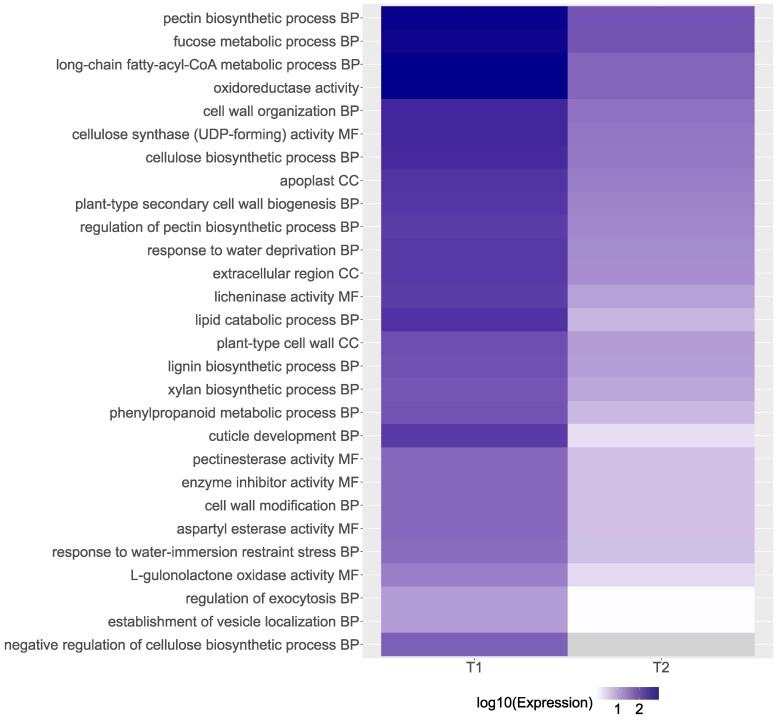
Heatmap of gene expression for top 28 significantly downregulated GO terms following drought treatment. The GO terms were enriched with genes at 2 time points, T1, at the initiation of soil drying treatment and T2, 16 d following treatment. Average expression was measured in 12 *Quercus douglasii* seedlings sampled at 2 time points by first taking the average expression for each gene for all individuals by maternal family, and then averaging values for all genes annotated with the given GO term. Log-fold changes varied from no expression (gray) to low (white) to −3 (dark blue) log_10_ decrease in mean expression. Only those GO terms with the greatest difference in expression are shown, see Supplementary Fig. 1 for a comprehensive list. Abbreviations correspond to GO terms: BP, biological process; CC, cellular component; MF, molecular function.

In contrast to the upregulated genes, we found a significant effect of maternal family on expression of downregulated genes (Supplementary Table 2). In addition, compared to our upregulated genes, we found 3-fold more genes that were downregulated (123 genes, Fig. 2b) in our drought-stressed individuals compared with their well-watered controls. In total, we found 71 enriched GO terms among downregulated genes (Supplementary Fig. 1), and show the top 28 GO terms (Fig. 4), which comprises genes with at least an 87% decrease in expression in the drought treatment between time periods. These most strongly downregulated GO terms largely corresponded to biological process functions, many of which were related to growth, such as “lipid catabolic process,” “cuticle development,” and various processes related to synthesis of complex sugars found in plant cell walls (Fig. 4). Enriched GO terms also related to the downregulation of reproductive processes, including “seed coat development.”

### Constitutive expression of drought-responsive genes

Given how few genes were up- or downregulated in *Q. douglasii*, we examined gene expression across time periods for the 81 “drought-responsive” Pfam categories selected (Supplementary Table 3) because they showed differentially expressed genes in 2 *Q. lobata* drought experiments (Gugger et al. 2017; Mead et al. 2019a). This list included several stress-responsive functions; for example, a universal stress protein family (PF00582), heat-shock protein 90 (PF00447 and PF02518), and an auxin response factor (PF06507). For both treatments, we found relatively high mean expression of these drought-responsive Pfams between time points (Supplementary Fig. 2) but levels of expression between time points were not significant (Supplementary Table 5), because they were being continuously expressed in both the drought and well-watered treatments. Average expression was significantly higher in the drought than well-watered treatment at both time points, although only slightly (Supplementary Fig. 2, Supplementary Table 5).

**Fig. 5.**
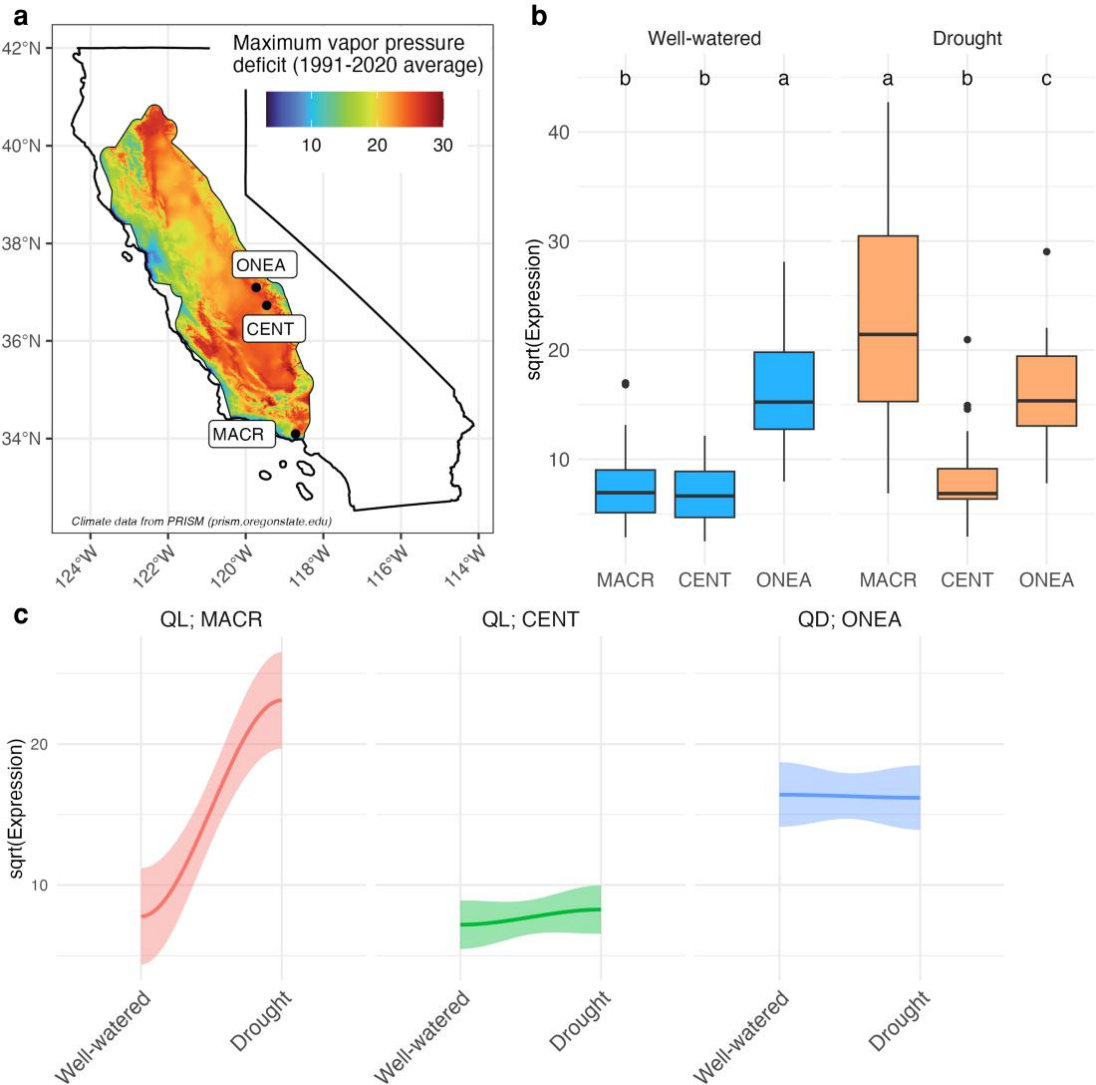
Gene expression for 20 drought-responsive protein families (Pfams) in *Quercus lobata* and *Q. douglasii* well-watered and drought treatments. a) Locations of sampled trees in California overlayed with maximum vapor pressure deficit (the difference between the amount of moisture in the air and how much moisture the air can hold at saturation, where higher values indicate drier sites) within the *Q. lobata* species range (1991 to 2020 average; PRISM, prism.oregonstate.edu). *Q. lobata* was sampled in Malibu Creek (MACR) and Centerville (CENT), and *Q. douglasii* acorns from O’Neals (ONEA). Vapor pressure deficit is lower in MACR as compared with CENT and ONEA. b) Mean expression under well-watered and drought treatments of 20 drought-responsive Pfams that were differentially expressed across the 2 *Q. lobata* sites (MACR and/or CENT) and present in the third site for *Q. douglasii* (ONEA). Expression was calculated by negative binomial generalized linear models in DESeq2 across well-watered and drought treatments for genes within each Pfam category with average values calculated from raw read counts across individuals. Letters represent significant differences between sites across each treatment (post hoc Tukey test with Bonferroni-adjusted *P*-values: expression × site, random effect = Pfam). c) Mean expression of 20 drought-responsive Pfams under well-watered and drought treatments by site. Expression was calculated as for b and with standard error per Pfam plotted. Mean expression of *Q. lobata* individuals at MACR show an effect in response to drought (slope = 0.84, *P* < 0.001) but no difference among *Q. lobata* individuals at CENT (slope = 0.17, *P* > 0.05) nor *Q. douglasii* individuals at ONEA (slope = 0.01, *P* > 0.05). The slopes significantly differ by both site and Pfam (ANOVA: site, *P* < 0.001; Pfam, *P* < 0.01). See Supplementary Table 4 for individual Pfam regression coefficients (slopes).

To further examine evidence of constitutive expression in *Q. douglasii*, we examined expression levels of a set of drought-responsive genes between 2 species. Specifically, we compared levels of genes matching 20 drought-responsive Pfams at T1 and T2 in the drought treatment with those from 2 localities reported for *Q. lobata* by Mead et al. (2019a). The 20 Pfams correspond to 80 differentially expressed genes in *Q. lobata* and 2,382 genes (and 3,731 transcripts) in *Q. douglasii.* In the well-watered treatment, *Q. douglasii* had the highest level of gene expression, and in the drought treatment, one of the *Q. lobata* sites from a cooler climate (Malibu Creek) had the highest expression level (Fig. 5b). Our analysis of how gene expression changes from well-watered to drought conditions revealed that *Q. douglasii* had the highest constitutive expression and further showed that drought treatment did not change the expression of these 20 drought-responsive Pfams in either *Q. douglasii* nor *Q. lobata* from similar hotter climates (O’Neals and Centerville) but did increase their expression in *Q. lobata* from a contrasting milder climate (Malibu Creek; Fig. 5b). *Q. douglasii* had the highest constitutive expression while *Q. lobata* at Malibu Creek had the most differential gene expression (Fig. 5c).

## Discussion

Our study illustrates a rarely reported response to drought stress—constitutive gene expression—exhibited by seedlings collected from a California population of a drought-tolerant oak, *Q. douglasii*. We found more downregulated genes than upregulated genes, and unexpectedly many drought-responsive genes that appear to be constitutively expressed. Downregulated genes were typically involved in growth, indicating that drought tolerance may come at the cost of growth. Few upregulated genes may be explained by the high number of drought-responsive genes that were already expressed at the start of the experiment. Results from our drought stress experiment highlight that *Q. douglasii* seedlings respond to drought both through gene expression plasticity triggered by low soil moisture and also through genes with constitutive expression. Thus, in a drought-adapted species exposed to frequent water stress, constitutive gene expression may be a key strategy of their adaptation to low water environments.

### Gene expression plasticity in *Q. douglasii*

The seedlings in this study showed few differentially expressed genes in response to drought, which could relate to the fact that *Q. douglasii* is a drought-tolerant species. Similar to our study, 2 independent studies in oaks in Europe and California (Madritsch et al. 2019; Mead et al. 2025) found that drought-tolerant oak species (*Q. ilex* and *Q. pubescens* in Europe; *Q. palmeri, Q. chrysolepis, Q. durata*, and *Q. agrifolia* in California) exhibited fewer differentially expressed genes than less drought-tolerant species (*Q. robur* in Europe; *Q. lobata* and *Q. kelloggii* in California) in response to soil dry-down treatment. We can gain some insight about why drought-tolerant species have fewer differentially expressed genes from the European study (Madritsch et al. 2019). The authors conclude that the 2 drought-tolerant Mediterranean species exhibited drought avoidance and the most drought-tolerant species differentially expressed many fewer genes. In contrast, *Q. robur* encountered severe stress, as evidenced by lowered root growth, which they interpret to be evidence of drought intolerance. Thus, it seems that drought-tolerant oaks may not need to respond to drought stress by increasing gene expression.

Gene functions for upregulated genes were similar to those reported previously for oaks (Gugger et al. 2017; Madritsch et al. 2019; Mead et al. 2019a), with most gene functions broadly associated with drought response. For example, serine acetyltransferase 1 was upregulated, an important component of the cysteine synthase complex, which in turn regulates the biosynthesis of abscisic acid (ABA), a major hormone known to mediate drought response. Various studies have reported the upregulation of serine acetyltransferases in response to abiotic stress (Kurt et al. 2021; Wang et al. 2024). Additionally, the ethylene-signaling protein RTE1 (REVERSION-TO-ETHYLENE SENSITIVITY 1) was upregulated, which reduces sensitivity to ethylene (another stress hormone) and improves drought tolerance when overexpressed in *Arabidopsis* and maize (Shi et al. 2015). Finally, 2 transcription factors were upregulated, a basic helix-loop-helix (bHLH) and an NAC transcription factor, which both have well-reported roles in plant response to abiotic stress, eg bHLH promotes drought tolerance in tomato by switching on genes encoding antioxidants, ABA-signaling molecules, and stress-related proteins (Liang et al. 2022) and NAC regulates endogenous ABA in *Arabidopsis* (Jensen et al. 2013). The preponderance of differential expression of drought-related genes indicates that soil-drying triggered genes that respond to drought stress.

In our study most differentially expressed genes were downregulated, a finding that is also reported in other plant species, such as 2 desert species (Long et al. 2014; Ma et al. 2015) and willows (Pucholt et al. 2015). In terms of downregulated gene functions, our findings were consistent with other studies that reported an inhibition of photosynthesis, carbohydrate metabolism, and cell division under drought stress (Hayano-Kanashiro et al. 2009; Gugger et al. 2017; Zhang et al. 2018). Carbohydrate metabolism and biosynthesis are critical plant processes for capturing energy produced during photosynthesis, and its substrates are known to play a role in responses to drought stress as well as providing energy. Carbohydrate levels in tissues can be changed by differential expression of the genes underlying carbohydrate metabolism and biosynthesis. We report the downregulation of 5 GO terms related to carbohydrate metabolism: rhamnogalacturonan I side chain metabolic process; long-chain fatty-acyl-CoA metabolic process; xylan metabolic process; fucose metabolic process; rhamnogalacturonan I metabolic process. We additionally report the downregulation of 10 GO terms related to carbohydrate biosynthetic processes: xylan biosynthetic process; lignin biosynthetic process; negative regulation of cellulose biosynthetic process; cellulose biosynthetic process; glucuronoxylan biosynthetic process; regulation of pectin biosynthetic process; pectin biosynthetic process; rhamnogalacturonan I biosynthetic process; mucilage pectin biosynthetic process; globoside biosynthetic process. Therefore, carbohydrate activities were significantly repressed under water restriction, which would result in a carbon deficiency. This response in turn would affect the chloroplast and cell wall and other important developmental functions. The preponderance of growth or metabolism processes shutting down with water stress in *Q. douglasii* could reflect a trade-off between growth and drought tolerance mechanisms.

Within our sample of seedlings from a single local population, we discovered that the downregulated genes exhibited variable gene expression in response to drought (treatment × time × maternal family interaction), while the upregulated genes did not. This finding illustrates variation within our population for gene expression plasticity, namely that we see no difference between maternal family in the upregulation of a small number of drought-responsive genes while differences in downregulated genes involved in growth could indicate variation in stress tolerance among maternal families. Rivera et al. (2021) define tolerance as survival or maintained organismal functioning under stress, and the magnitude of this downregulation affecting how well each *Q. douglasii* family can maintain growth could be a factor contributing to tolerance within each family. Further understanding of the impact of drought stress on growth will require larger sample sizes of families and seedlings within families. The effectiveness of the drought response may depend on how plants perceive drought stress signals and respond via changes in gene expression.

### Constitutive gene expression as a drought response strategy

Our finding that many drought-responsive genes in *Q. douglasii* were not differentially expressed and instead showed high baseline expression across both well-watered and drought treatments compared with *Q. lobata* suggests different drought response strategies across the 2 species. Namely, the lack of a transcriptional response to drought treatment in the more drought-tolerant *Q. douglasii* was due to high constitutive expression, or front-loading, of drought-responsive genes compared with low or differential expression in the less drought-tolerant *Q. lobata.* This strategy could at least partly contribute to the species' drought-tolerant nature, and reinforces the value of analyzing constitutive expression in individuals sampled from multiple populations.

Constitutive gene expression in *Q. douglasii* appears to be an adaptation to limited water availability. This interpretation is supported by a separate gene expression study across 6 California oak species ranging in drought tolerance (Mead et al. 2025), in which higher constitutive expression was reported for a subset of genes in drought-tolerant oak species. Together, these findings highlight that drought response could result from plasticity in gene expression, or local adaptation for constitutive gene expression. Gene expression links genotype to adaptive phenotypes, making gene expression plasticity an important functional response to environmental change across generations (De Nadal et al. 2011). However, our findings highlight that another rarely explored yet potentially important component of functional response to drought could be continuous gene expression.

## Conclusion

*Q. douglasii* seedlings at this Sierra Mountain foothill population exhibit higher constitutive expression of drought-responsive genes compared with the drought-sensitive *Q. lobata.* In a drought-tolerant species, water limitation could be less stressful thanks to frontloading, in which higher constitutive expression of specific genes promotes tolerance to stress by maintaining homeostasis and cellular integrity. In contrast, a drought-sensitive species may show lower baseline expression of drought-responsive genes because it may experience this type of stress less frequently, resulting in a strong gene expression plasticity response when water limitation is encountered. We identified drought-responsive protein families that underlie drought stress response in both *Q. douglasii* and *Q. lobata,* albeit via different patterns of gene expression. This study is unique in its discovery of constitutive gene expression as a potential adaptation for tolerance to drought stress in any tree species, illustrating that plasticity in gene expression is not the only response strategy that will allow trees to tolerate drought stress.

## Supplementary Material

jkaf293_Supplementary_Data

## Data Availability

All raw sequence data have been deposited in the relevant International Nucleotide Sequence Database Collaboration (INSDC) database with the BioProject ID PRJNA1259526. The annotated *Q. douglasii* transcriptome analyzed in this manuscript is published in a publicly available Zenodo digital repository (10.5281/zenodo.17793755), along with source data, differential expression output files, custom analysis and plotting scripts. We identified drought-responsive genes using supplementary data files in Gugger et al. (2017) and Mead et al. (2019a), and additionally we re-analyzed publicly available data (Mead et al. 2019b). Supplemental material available at G3 online.
