# Supplementary material for: Drought-associated genes exhibit high constitutive expression in Quercus douglasii, a drought-tolerant California oak
Source: G3 (Bethesda). 2025 Dec 12;16(2):jkaf293. doi: 10.1093/g3journal/jkaf293 (PMC12869068; doi:10.1093/g3journal/jkaf293)
Supplement: jkaf293_Supplementary_Data [file jkaf293_supplementary_data.pdf]

Supplementary Material

for

Drought-associated genes exhibit high constitutive expression in *Quercus douglasii*, a drought-tolerant California oak

Stephanie E. Steele[1^]

Lily D. Peck[1 ^]

Victoria L. Sork[1,2 *]

[1] Department of Ecology and Evolutionary Biology, University of California, Los Angeles, California, 90095-7239, USA

[2] Institute of the Environment and Sustainability, University of California, Los Angeles, California, 90095-1496 USA

^ Equal contribution, co-first authors.

*Corresponding author: VL Sork, Department of Ecology and Evolutionary Biology, University of California Los Angeles, 610 Charles E. Young Drive East, Los Angeles, CA 90095-7239, United States, Email: vlsork@ucla.edu

Supplementary Tables

Table S1. Results of ANOVA for up-regulated genes, comparing an effect of treatment, time, and maternal family on gene expression in *Q. douglasii* seedlings. Mixed-effects model: expression ~ treatment | time | family, random effect = gene. Abbreviations: num., numerator; den., denominator.

	Degrees of freedom (num.)	Degrees of freedom (den.)	*F*-value	P-value	Adjusted P-value	Significance
(Intercept)	1	1340	12.89	3.42E-04	1.37E-03	*
Treatment	1	1340	4.11	4.30E-02	8.59E-02	ns
Time	1	1340	58.50	3.86E-14	3.09E-13	*
Family	5	1340	1.34	2.45E-01	2.80E-01	ns
Treatment x Time	1	1340	6.18	1.30E-02	3.48E-02	*
Treatment x Family	5	1340	1.44	2.08E-01	2.77E-01	ns
Time x Family	5	1340	0.74	5.91E-01	5.91E-01	ns
Treatment x Time x Family	5	1340	2.04	7.11E-02	1.14E-01	ns

Table S2. (**A)**. Results of ANOVA for down-regulated genes, comparing an effect of treatment, time, and maternal family on gene expression in *Q. douglasii* seedlings. Mixed effects model: expression ~ treatment | time | family, random effect = gene. Abbreviations: num., numerator; den., denominator. (**B)**. Results of a post-hoc Tukey test comparing pairwise maternal family effects. ANOVA: expression ~ family + (1+gene). The maternal families are QUDO_1, QUDO_2, QUDO_5, QUDO_7, QUDO_9 and QUDO_10.

A	Degrees freedom (num.)	Degrees freedom (den.)	F-value	P-value	Adjusted P-value	Significance
(Intercept)	1	5758	54.91	1.44E-13	2.89E-13	*
Treatment	1	5758	1.17	2.79E-01	2.79E-01	ns
Time	1	5758	373.98	<2.20E-16	<2.20E-16	*
Family	5	5758	24.59	<2.20E-16	<2.20E-16	*
Treatment x Time	1	5758	20.95	4.81E-06	5.50E-06	*
Treatment x Family	5	5758	27.79	<2.20E-16	<2.20E-16	*
Time x Family	5	5758	6.61	3.83E-06	5.10E-06	*
Treatment x time x Family	5	5758	8.85	2.23E-08	3.57E-08	*

B. Pairwise contrast between families.

Mat. Fam. 1	Mat. Fam. 2	Null. value	Estimate	Std. error	T-statistic	Adjusted P-value	Significance
QUDO_10	QUDO_1	0	-10.5	4.52	-2.32	0.309	
QUDO_2	QUDO_1	0	-14.4	4.52	-3.18	0.0229	*
QUDO_5	QUDO_1	0	5.6	4.52	1.24	1	
QUDO_7	QUDO_1	0	-16.9	4.52	-3.74	0.00292	**
QUDO_9	QUDO_1	0	-15.9	4.52	-3.51	0.00696	**
QUDO_2	QUDO_10	0	-3.9	4.52	-0.86	1	
QUDO_5	QUDO_10	0	16.1	4.52	3.56	0.00572	**
QUDO_7	QUDO_10	0	-6.4	4.52	-1.42	1	
QUDO_9	QUDO_10	0	-5.4	4.52	-1.19	1	
QUDO_5	QUDO_2	0	20.0	4.52	4.42	0.000161	***
QUDO_7	QUDO_2	0	-2.5	4.52	-0.56	1	
QUDO_9	QUDO_2	0	-1.5	4.52	-0.33	1	
QUDO_7	QUDO_5	0	-22.5	4.52	-4.98	0.0000108	***
QUDO_9	QUDO_5	0	-21.5	4.52	-4.75	0.0000334	***
QUDO_9	QUDO_7	0	1.0	4.52	0.23	1	

Table S3. A list of 81 protein families (Pfams) that were differentially expressed in two drought studies of a drought sensitive oak, *Quercus lobata* (P < 0.05 in Gugger et al., 2017[1], adjusted P-value < 0.01 in Mead et al., 2019[2]), and were examined for gene expression in *Q. douglasii*. The regression coefficient values for the 81 drought-responsive protein families (Pfams) in *Q. douglasii* across Time 1 and Time 2 of well-watered and drought treatments. Coefficient values for the effect of time within each Pfam were calculated using the linear model "expression ~ time * Pfam" in R.

Protein family (Pfam)	Protein family name	Coefficient values	
		Well-watered	Drought
PF01073	3-beta hydroxysteroid dehydrogenase/isomerase family	0.148	0.047
PF00005	ABC transporter	-0.021	-0.002
PF00664	ABC transporter transmembrane region	0.001	0.019
PF01842	ACT domain	0.032	0.034
PF00578	AhpC/TSA family	0.122	-0.011
PF01918	Alba	-0.014	-0.014
PF07859	alpha/beta hydrolase fold	-0.065	-0.024
PF00847	AP2 domain	-0.156	-0.127
PF02309	AUX/IAA family	-0.008	-0.013
PF06507	Auxin response factor	-0.008	-0.016
PF02362	B3 DNA binding domain	-0.032	-0.029
PF07716	Basic region leucine zipper	-0.113	-0.088
PF00170	bZIP transcription factor	-0.106	-0.083
PF00149	Calcineurin-like phosphoesterase	-0.011	-0.009
PF06203	CCT motif	-0.183	-0.153
PF00125	Core histone H2A/H2B/H3/H4	-0.624	-0.386
PF02485	Core-2/I-Branching enzyme	0.030	0.012
PF02984	Cyclin, C-terminal domain	0.016	0.004
PF01529	DHHC palmitoyltransferase	0.037	0.029
PF00609	Diacylglycerol kinase accessory domain	0.039	0.037
PF00781	Diacylglycerol kinase catalytic domain	0.000	-0.001
PF00122	E1-E2 ATPase	-0.047	-0.035
PF13405	EF-hand domain	-0.069	-0.073
PF03789	ELK domain	-0.555	-0.563
PF12937	F-box-like	-0.017	-0.019
PF01565	FAD binding domain	0.113	0.049
PF00254	FKBP-type peptidyl-prolyl cis-trans isomerase	0.041	0.030

[1] Gugger, P.F., J.M. Peñaloza-Ramírez, J.W. Wright, and V.L. Sork, 2017 Whole-transcriptome response to water stress in a California endemic oak, *Quercus lobata*. *Tree Physiology* 37 (5):632-644

[2] Mead, A., J. Peñaloza Ramirez, M.K. Bartlett, J.W. Wright, L. Sack, and V.L. Sork, 2019 Seedling response to water stress in valley oak (*Quercus lobata*) is shaped by different gene networks across populations. *Molecular Ecology* 28 (24):5248-5264

Protein family (Pfam)	Protein family name	Coefficient values	
		Well-watered	Drought
PF07777	G-box binding protein MFMR	-0.048	-0.071
PF00462	Glutaredoxin	-0.017	0.011
PF13409	Glutathione S-transferase, N-terminal domain	-0.028	-0.003
PF13417	Glutathione S-transferase, N-terminal domain	-0.011	0.017
PF00232	Glycosyl hydrolase family 1	-0.025	0.110
PF01501	Glycosyl transferase family 8	-0.097	-0.153
PF00534	Glycosyl transferases group 1	0.021	0.063
PF00010	Helix-loop-helix DNA-binding domain	-0.008	0.011
PF02518	Histidine kinase-, DNA gyrase B-, and HSP90-like ATPase	0.074	0.153
PF00808	Histone-like transcription factor (CBF/NF-Y) and archaeal histone	-0.399	-0.232
PF02183	Homeobox associated leucine zipper	-0.006	-0.034
PF05920	Homeobox KN domain	-0.218	-0.169
PF00046	Homeodomain	-0.016	-0.024
PF00447	HSF-type DNA-binding	-0.061	-0.050
PF00183	Hsp90 protein	0.454	0.576
PF03790	KNOX1 domain	-0.257	-0.410
PF03791	KNOX2 domain	-0.257	-0.410
PF00560	Leucine Rich Repeat	0.005	-0.002
PF13855	Leucine rich repeat	0.001	-0.008
PF07690	Major Facilitator Superfamily	-0.018	-0.019
PF00249	Myb-like DNA-binding domain	-0.045	-0.035
PF02719	NA	0.252	0.114
PF03131	NA	-0.057	-0.040
PF01370	NAD dependent epimerase/dehydratase family	0.074	0.025
PF02365	No apical meristem (NAM) protein	-0.031	-0.041
PF00956	Nucleosome assembly protein (NAP)	-0.111	-0.052
PF00293	NUDIX domain	0.005	0.001
PF00141	Peroxidase	0.136	0.049
PF00658	Poly-adenylate binding protein, unique domain	-0.127	0.010
PF00854	POT family	0.007	0.006
PF00069	Protein kinase domain	-0.012	-0.018
PF07714	Protein tyrosine kinase	-0.012	-0.017
PF01412	Putative GTPase activating protein for Arf	0.001	-0.010
PF00070	Pyridine nucleotide-disulphide oxidoreductase	0.096	0.063
PF07992	Pyridine nucleotide-disulphide oxidoreductase	0.042	0.027
PF08534	Redoxin	0.128	-0.005
PF01694	Rhomboid family	0.043	0.012

Protein family (Pfam)	Protein family name	Coefficient values	
		Well-watered	Drought
PF00237	Ribosomal protein L22p/L17e	0.018	0.046
PF04321	RmlD substrate binding domain	0.342	0.191
PF00076	RNA recognition motif. (a.k.a. RRM, RBD, or RNP domain)	-0.032	-0.023
PF05773	RWD domain	-0.063	-0.037
PF02383	SacI homology domain	-0.016	0.009
PF08501	Shikimate dehydrogenase substrate binding domain	0.243	0.452
PF00083	Sugar (and other) transporter	-0.011	-0.018
PF00515	Tetratricopeptide repeat	0.012	0.006
PF13181	Tetratricopeptide repeat	0.004	0.002
PF02458	Transferase family	0.035	0.038
PF02358	Trehalose-phosphatase	-0.008	-0.042
PF01487	Type I 3-dehydroquinase	0.222	0.407
PF00179	Ubiquitin-conjugating enzyme	-0.025	-0.021
PF00201	UDP-glucoronosyl and UDP-glucosyl transferase	-0.007	-0.008
PF00582	Universal stress protein family	-0.025	-0.017
PF00400	WD domain, G-beta repeat	-0.027	-0.010
PF00096	Zinc finger, C2H2 type	-0.052	-0.053

Table S4. The regression coefficient values for 20 drought-responsive protein families (Pfams) that were differentially expressed in *Quercus lobata* in both Mead et al. (2019) and Gugger et al. (2017) and that comprise at least two differentially expressed genes. Values are shown across two *Q. lobata* sites (Malibu Creek, MACR and Centerville, CENT) and one *Q. douglasii* site (O'Neals, ONEA). In R, values were calculated using the linear mixed effects model: expression ~ time + pfam, random effect = gene name.

Pfam	QL; MACR	QL; CENT	QD; ONEA
PF13855	-0.885	0.025	3.41E-04
PF00249	-0.827	0.037	0.144
PF00141	0.080	-0.156	0.872
PF02458	-0.416	-0.063	0.040
PF00069	-0.789	0.033	0.014
PF07714	-0.698	-0.149	0.014
PF02365	-0.808	-0.321	0.119
PF00076	-0.777	0.347	0.536
PF00201	-0.665	0.671	0.257
PF07859	-0.314	-0.088	0.306
PF00462	-0.770	0.276	0.267
PF00664	-0.638	-0.029	0.566
PF00005	-0.375	NA	0.567
PF00854	NA	0.826	0.134
PF00534	0.538	1.484	0.730
PF01501	-0.476	0.597	1.488
PF00122	-0.959	-0.278	0.678
PF01487	-0.246	-0.151	0.389
PF08501	-0.246	-0.151	0.390
PF00560	-1.030	-0.014	NA

Table S5. Results of ANOVA for gene expression at both timepoints between drought and well-watered treatments, comparing an effect of treatment and time on gene expression in *Q. douglasii* seedlings. ANOVA: expression ~ treatment | time, random effect = Pfam, run in R. Abbreviations: num., numerator; den., denominator.

	**Degrees freedom (num.)**	**Degrees freedom (den.)**	**F-value**	**P-value**
(Intercept)	1	51528	184.6056	<.0001
Treatment	1	51528	23.6629	<.0001
Time	1	51528	1.0615	0.3029
Treatment x Time	1	51528	0.0512	0.8210

Supplementary Figures

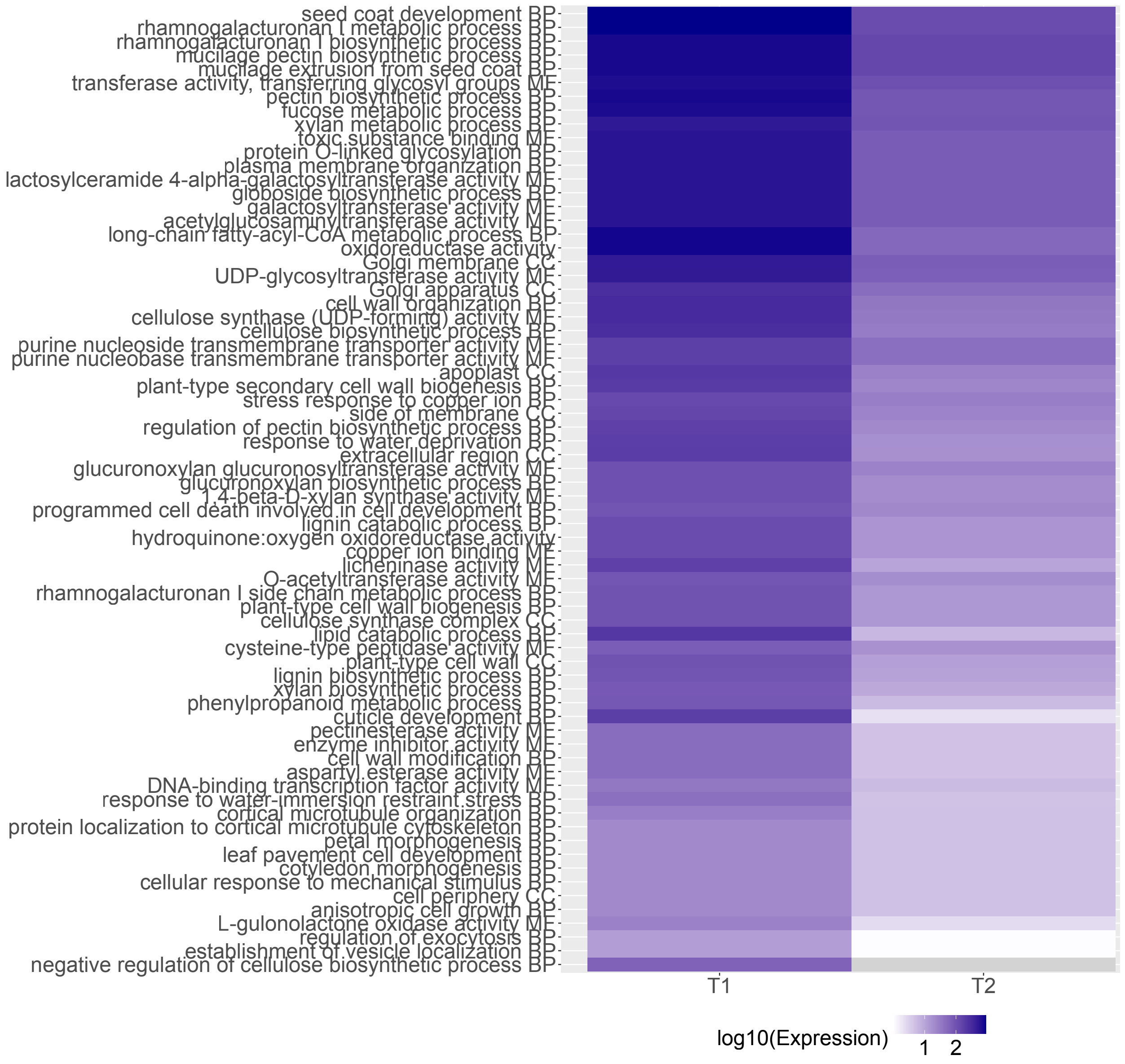

Figure S1. Heatmap of mean gene expression for 71 significantly down-regulated gene ontology (GO) terms. The GO terms were enriched with genes at two time points, T1, at the onset of soil drying treatment and T2, sixteen days following treatment. Average expression was measured in 12 *Quercus douglasii* seedlings sampled at two time points in the drought treatment group by first taking the average expression for each gene for all individuals by maternal family, and then averaging values for all genes annotated with the given GO term. Log-fold changes varied from no expression (grey) to low (white) to -3 (blue) log10 decrease in mean expression. Abbreviations correspond to GO terms: BP, biological process; CC, cellular component; MF, molecular function.

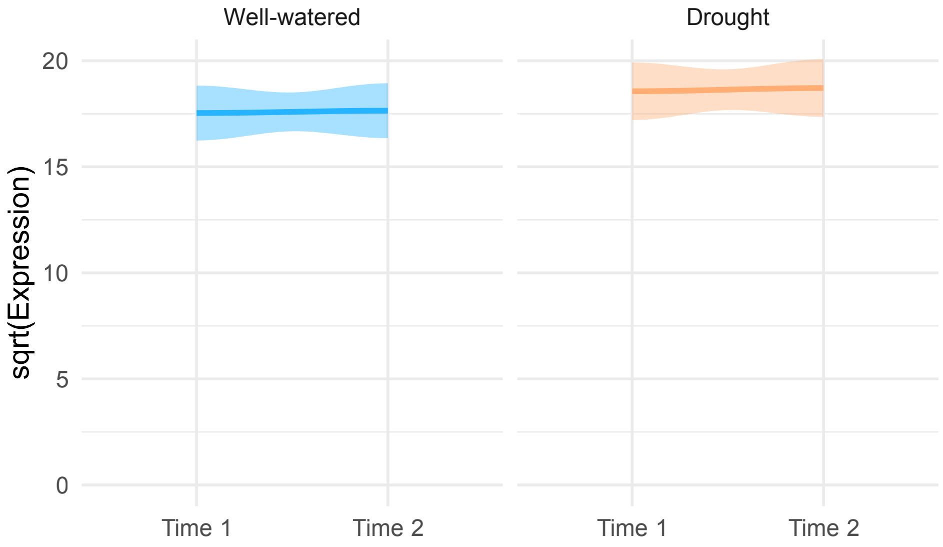

Figure S2. Gene expression for 81 drought-responsive protein families (Pfams) in *Quercus douglasii* drought and well-watered treatments. Each line represents the mean expression and standard error (estimated using negative binomial generalized linear models in DESeq2) of genes across Time 1 and Time 2. Genes were represented by 15,016 transcripts. Average expression across all 81 Pfams did not change between timepoint in either treatment (drought: slope = 0.007, $P > 0.05$; well-watered: slope = 0.01, $P > 0.05$), see Table S4 for individual Pfam regression coefficient (slope) values. Average expression across all 81 Pfams at both time points was significantly (and slightly) higher in drought than well-watered treatments (ANOVA, $p < 0.0001$, see Table S5).
